# Dissociating the Role of the pre-SMA in Response Inhibition and Switching: A Combined Online and Offline TMS Approach

**DOI:** 10.3389/fnhum.2013.00150

**Published:** 2013-04-18

**Authors:** Ignacio Obeso, Noemí Robles, Elena M. Marrón, Diego Redolar-Ripoll

**Affiliations:** ^1^Cognitive Neuro-Lab, Cognitive Neuroscience and Information Technologies Research Program, IN3, Open University of CataloniaBarcelona, Spain; ^2^Reward and Decision Making Group, Cognitive Neuroscience Center, CNRS, Lyon 1 UniversityLyon, France

**Keywords:** response inhibition, switching, working memory, cognitive control, TMS, pre-SMA

## Abstract

The pre-supplementary motor area (pre-SMA) is considered to be a key node in the cognitive control of actions that require rapid updating, inhibition, or switching, as well as working memory. It is now recognized that the pre-SMA is part of a “cognitive control” network involving the inferior frontal gyrus (IFG) and subcortical regions, such as the striatum and subthalamic nucleus. However, two important questions remain to be addressed. First, it is not clear if the main role of the pre-SMA in cognitive control lies in inhibition or switching of actions. From imaging evidence, the right pre-SMA is consistently recruited during inhibition and switching, but the extent to which it participates specifically in either of these processes is unknown. Secondly, the pre-SMA may perform inhibition and switching alone or as part of a larger brain network. The present study used *online* and *offline* transcranial magnetic stimulation (TMS) to dissociate the roles of pre-SMA in cognitive control, but also to investigate the potential contribution of connectivity between the pre-SMA and IFG. We applied continuous theta burst stimulation (cTBS) over the right IFG before participants performed a stop switching task while receiving single TMS pulses over the right pre-SMA. The results were compared to a sham cTBS session and pulses applied over the vertex region. Significant worsening of inhibition as well as response adaptation during inhibition was found when applying pulses over the pre-SMA. However, no such worsening was observed in switch trials. Additionally, after cTBS over the IFG, inhibition was also delayed, suggesting its critical necessity in stopping of actions. The results reveal a key contribution of the pre-SMA in inhibition and could suggest a dissociative role in the switching of actions. These findings indicate there is an essential union between IFG and pre-SMA during inhibition.

## Introduction

Visualize yourself running to catch your regular bus back home. All of a sudden, someone gets in the way. You can either stop running, or change direction to avoid crashing into that person. Depending on several variables (i.e., your speed, distance to the person, motivation to catch the bus), you may need to select one of several possible actions. During the rapid updating of actions, such as in this example, one needs to abort action altogether, or implement new actions.

Several processes relating to cognitive control are important for this behavior. Reactive and proactive inhibition, switching of actions, and working memory, are key functions required to resolve sudden conflict. In the above example, one could stop entirely when the person gets in your way, as a reaction to the rapid and unexpected change in the context (reactive inhibition). However, one could also pre-emptively act so as to not run too fast on the way to the bus stop, as the probability of encountering a bypassing pedestrian in the street are often high, therefore this approach would almost guarantee a successful outcome (proactive inhibition and working memory). There is also the option of switching to new actions, such as the decision to run in a different direction and avoid crashing. In sum, these three cognitive control processes are used in parallel (Nachev et al., 2008) to avoid unwanted actions and achieve goal-directed behavior.

These behaviors can be measured experimentally. To measure inhibition of actions, a task called stop signal reaction time (SSRT) task is often used. In brief, participants are required to respond to go and stop stimuli. During Go trials, participants respond as quickly as possible to, for example, a right or left pointing arrow. A proportion of the Go trials are followed by a stop signal that requires participants to inhibit their movement. The stop signal is presented after an adaptive delay that reflects each participants’ inhibitory ability. Additional conditions can also evaluate switching or context adaptation of actions.

The above types of behaviors are part of a cognitive control mechanism that is implemented in the brain through a prefrontal-basal ganglia circuit. Key areas in cognitive control are the pre-supplementary motor area (pre-SMA), inferior frontal gyrus (IFG), and some regions of the basal ganglia, such as caudate or the subthalamic nucleus (STN), as revealed by functional magnetic resonance imaging (fMRI) evidence (Rubia et al., 2001; Rushworth et al., 2002; Aron and Poldrack, 2006; Aron et al., 2007; Li et al., 2008; Kenner et al., 2010; Zandbelt and Vink, 2010). In addition, the contribution of the dorsolateral prefontal cortex (DLPFC) in situations with greater cognitive demand, i.e., more working memory load, is also of special interest in the framework of cognitive control (Mostofsky et al., 2003; Jahfari et al., 2010; Criaud and Boulinguez, 2012). However, the contribution of each region in changing or adaptation of behavior is still largely uncertain. One repetitive transcranial magnetic stimulation (rTMS) study reported that the function of the IFG can be subdivided such that the pars triangularis region is involved in updating of action plans, while a more dorsal region of the IFG is important for target detection (Verbruggen et al., 2010). Also, disrupting right IFG activity using repetitive TMS altered the speed of inhibition during a stop signal task combined with flankers (Chambers et al., 2007). Therefore, the IFG could be seen as a multifunctional hub responsible for different processes associated to response inhibition. So far, a causal relationship has not been established for the IFG with respect to switching-related behavior.

Regarding the role of the pre-SMA in response inhibition, greater activity in this region during trials that are successfully stopped compared to failed inhibition has been observed (Aron et al., 2007; Chevrier et al., 2007; Chikazoe et al., 2009; Duann et al., 2009; Boehler et al., 2010; Hampshire et al., 2010; Sharp et al., 2010; Cai and Leung, 2011; Tabu et al., 2011). Pre-SMA lesions have also confirmed the important role of this region in stopping actions (Floden and Stuss, 2006; Nachev et al., 2007). The modulation of behavior when expecting a stopping stimulus (proactive inhibition) is a proposed function attached to the pre-SMA (Forstmann et al., 2008; Boulinguez et al., 2009; Chikazoe et al., 2009; Jahfari et al., 2010; Zandbelt and Vink, 2010), although some hypotheses posit the DLPFC as a candidate for proactive inhibition due to the working memory component in such behavior (Mostofsky et al., 2003; Jahfari et al., 2010; Aron, 2011; Criaud and Boulinguez, 2012). TMS has provided causal evidence for the pre-SMAs’ role in reactive inhibition of actions (Chen et al., 2009; Neubert et al., 2010; Verbruggen et al., 2010; Obeso et al., 2011, 2013; Cai et al., 2012), but none exists for proactive inhibition. In addition, switching from repetitive movements to new ones became worse by disrupting pre-SMA activity (Rushworth et al., 2002). Thus, the pre-SMA appears to be recruited during response inhibition and also during switching of actions.

Although both IFG and pre-SMA are critical in cognitive control, their interaction while resolving new behaviors is unknown. Is the IFG detecting critical stimuli, then triggering the activation of the pre-SMA? Or is the pre-SMA sending inputs to the IFG to be transmitted to subcortical regions? Using computational models to test causality in fMRI data (Duann et al., 2009; Jahfari et al., 2011), some have attempted to decipher how fronto-striatal interactions operate during response inhibition, suggesting that pre-SMA and M1 share a functional interconnectivity together with the basal ganglia during inhibition.

The aim of the current study was twofold: (i) to investigate the pre-SMAs’ role in response inhibition (both reactive and proactive), and/or switching, and (ii) to investigate frontal connectivity and the contribution of the right IFG during inhibition and switching of actions. The task used is a modified version of the stop signal task (Logan et al., 1984), and asked participants to respond as fast and accurate as possible to arrows in left and rightward directions. Both left and rightward arrows were presented alone as go trials. In the stop condition, the arrow was followed by an infrequent cross which indicated participants must stop any movement. In the switch condition, the arrow turns blue, requiring switching to a new movement. Before the task we applied inhibitory continuous theta burst stimulation (cTBS) over the right IFG or sham cTBS over M1. To see the influence of right IFG in pre-SMA functioning, following cTBS, participants performed the stop switching task while receiving TMS pulses (single pulse 100 ms after stimulus) over the right pre-SMA or vertex (control condition). We hypothesized that TMS pulses over the pre-SMA (after sham cTBS), compared to pulses over the vertex, would disturb inhibition and switching behavior, as well as the interaction between stop and switching behavior (proactive inhibition). Also, we predicted that IFG cTBS would impair inhibition of actions as compared to sham cTBS, showing a critical contribution of both areas during cognitive control.

## Materials and Methods

### Participants

Sixteen healthy right-handed volunteers (7 male), aged 24–44 years (*M* = 35.40, SD = 7.7) participated after meeting the TMS safety criteria (Rossi et al., 2009). None of them were taking any medication or had previous or actual neurological disorder or history of psychiatric illness, drug, or alcohol abuse. The study was approved by the Ethics Committee of the Universitat Oberta de Catalunya. Informed consent was obtained from all participants.

### Paradigm

**Figure 1:**
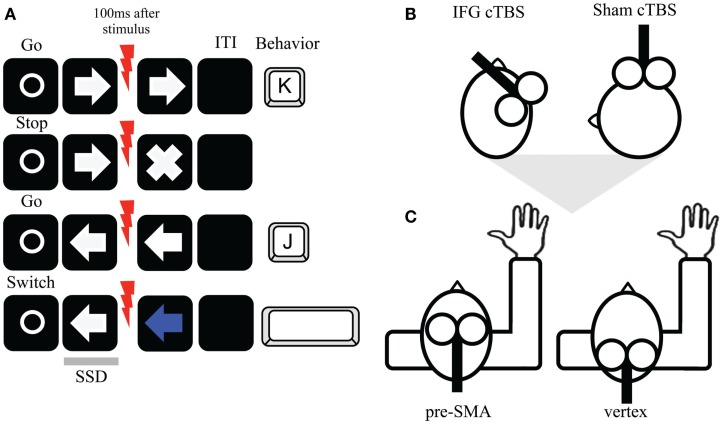
**(A)** Stop switching paradigm and timing of TMS pulses. On each trial, a fixation point was presented followed by a leftward or rightward arrow (go trial). Participants were asked to press as quickly as possible the “J” or “K” respectively. Occasionally, on the stop condition (right direction here), a white cross could appear (stop trial). Participants had to try to withhold their response, although they were aware that not always would be possible. In contrast, on the switch direction (left direction), sometimes the arrow changed to blue (switch trial) and participants tried to generate a new response by pressing the spacebar with their thumb finger. Single pulse TMS was given 100 ms after each trial type on half of the trials. **(B)** cTBS was applied firstly either over the right IFG or as sham over M1 (coil tilted 90°). **(C)** Following cTBS, participants performed the task while receiving single TMS pulses over the right pre-SMA or vertex (control condition).

We used a stop switching task (Figure 1A) modified from the stop signal paradigm (Logan et al., 1984). Participants used their right-dominant hand to respond via a computer keyboard. E-prime software was used for stimuli presentation (PC 15′, 85 Hz). On each trial, a white circle (fixation; 500 ms) indicated the start of a new trial followed by either a left or right pointing white arrow (go signal). The go signal indicated to respond as fast as possible with their right index (“J” key) and middle (“K” key) fingers, respectively. In the stop condition, after the white arrow (go signal) a white cross could appear (stop signal) shortly after the arrow was presented. Participants had to try to stop their already initiated response. This was applicable to the stop condition (e.g., right arrow direction). However, in the opposite switch condition, if the white arrow was presented in the opposite direction (e.g., left arrow direction), the arrow turned blue in some occasions. Participants had to try to generate a new response by pressing the space bar with their thumb finger. The arrow directions were counterbalanced across participants but also within participants. In the stop condition, the delay inserted between go and stop trials (stop signal delay, SSD) was based on a staircase tracking procedure, adjusted according to each participant response in stop trials. Three SSDs were initially set to 150 ms (SSD1), 200 ms (SSD2), and 250 ms (SSD3) and this was continuously adjusted to obtain approximately a 50% probability of inhibition. To achieve this, the SSD increased by 50 ms for each successful inhibition and decreased by 50 ms each time inhibition failed (Band et al., 2003). Participants were instructed not to wait for the stop signal to occur and that it would not always be possible to stop. The staircase method was also applied for the switch condition, although in reverse staircase steps, with 50 ms increase for successful switch trials and decrease by 50 ms each time switching failed. The limited response hold was 1 s. The intertrial interval (ITI) varied between 1 and 2.5 s. In total, there were four blocks of trials consisting of 72 go, 18 stop, and 18 switch trials per block (108 total trials per block).

### TMS thresholds

A Magstim super-rapid stimulator (Magstim, Whitland, Dyfed, UK) was used to stimulate cortical regions. We used a 7 cm-figure of eight-coil placed tangentially over the participant’s right M1 with 45° backwards and laterally over the hot-spot of the left hand first dorsal interosseous (FDI). The coil was first placed 2 cm anterior and 4 cm lateral to Cz (according to 10/20 EEG system) and repositioned where largest MEPs could be recognized and the hot-spot was marked on the participants scalp with a red marker. MEPs were registered using surface electrodes placed over participants’ left FDI. Resting motor thresholds (RMT) were defined over the hot-spot as the minimal stimulus intensity required to produce MEPs of ≥200 μV amplitude in ≥5 of 10 consecutive pulses. To obtain active motor thresholds (AMT), used in cTBS for safety reasons, muscle force was generated by squeezing a rubber band between the participants’ forefinger and thumb to activate their FDI muscle at approximately 10% of maximum force.

### MRI and target localization

We obtained a high-resolution T1 structural MRI from each participant at the Hospital de Mollet, Barcelona [3D FSPGR: slice thickness, 1 mm; repetition time (TR), 500 ms; echo time (TE), 50 ms; matrix, 256 × 256; field of view (FOV), 256; 180 sagittal slices].

Scans were used to localize each individual right IFG, right pre-SMA, and vertex targets. For IFG, we considered several fMRI studies of response inhibition (Aron et al., 2007; Chambers et al., 2007; Verbruggen et al., 2010), and found a convergence brain activation according to the following MNI coordinates *x* = 53, *y* = 24, *z* = 44 for the nearest voxel of the scalp surface. This point was marked on a cap using neuronavigation to localize it, for later application of cTBS without neuronavigation due to the physical obstacle of brainsight tracking system. For the pre-SMA, we selected a voxel of maximal activation from Aron et al. (2007), *x* = 6, *y* = 20, *z* = 44. For vertex (control target), we selected the intersection between the midline and the central sulci. For both online TMS conditions, the stimulation was delivered during real-time navigation system. Finally, for M1 sham stimulation we used the hot-spot as reference. Before stimulation, targets were localized using Brainsight frameless stereotaxic system (Rogue Research, Montreal Canada) with a Polaris (NorthernDigital, Waterloo, ON, Canada) infrared tracking system.

### Offline and online TMS

Once the target areas were localized, we applied inhibitory cTBS over the right IFG or sham over the right M1 (*offline stimulation*). Each train of cTBS (*offline TMS*) consisted of three pulses at 50 Hz, which is known to deactivate the stimulated neurons for a period of 40 min (Huang et al., 2005). Every cTBS burst was repeated at a 5 Hz rate resulting in 200 bursts with a total of 600 pulses at 80% of the AMT of each participant, lasting in total 40 s (Wassermann et al., 1996). For IFG stimulation, the coil was placed with the handle in an upward vertical orientation. For sham M1 stimulation, the coil was tilted 90° to mimic the sound of pulses as in the IFG TBS condition.

Upon termination of cTBS (Figure 1B), we stimulated the pre-SMA or vertex while participants performed the task (Figure 1C) approximately 2 min after cTBS termination. The *online* TMS approach was used to deliver 60% of maximum machine stimulation output. This fixed stimulation level was used in both regions as a standard intensity in frontal *online* stimulation (Rushworth et al., 2002; Ellison and Cowey, 2007; Chen et al., 2009) and keeps consistency across subjects. In both stimulations, the handle was oriented in a posterior direction inducing currents along the posterior-to-anterior axis (Figure 1C). No participants reported major adverse effects after *online* TMS pulses. In each block, a single TMS pulse (1.2 T; less than 1 ms duration) was delivered 100 ms after stimulus presentation for half of the trials in every trial type. The remaining trials were performed without TMS pulse (no pulse trials). TMS pulses were determined based on a previous TMS study using a similar approach (Chen et al., 2009). Separate SSD staircases were used for pulse and no pulse trials in both cTBS sessions.

### Experimental procedure

The experiment consisted on three separate days. On day 1, participants obtained a structural MRI. On days 2 and 3, after obtaining participants’ AMT, they initially received cTBS (over the right IFG) or sham cTBS (over the motor area). Then, the task was performed twice: once whilst receiving pulses over the right pre-SMA and once receiving pulses over the vertex. The order of cTBS was counterbalanced across participants, while the *online* TMS pulses were counterbalanced within participants. There was at least 1-week interval between each cTBS condition. A minimum practice of 40 trials was performed by every participant before cTBS application, with unlimited practice time until they verbally reported enough confidence with the instructions and task procedure.

### Main measures and analysis

A within-subject design was used to compare behavioral changes due to TMS pulses and cTBS effects on a stop switching task. We estimated SSRT using the integration method (Band et al., 2003). Other methods, such as the tracking procedure, assume that probability of inhibition will be close to 50% of the times. Here, examination of each participant’s data showed that this criterion was not met (*M* = 55.4%, SD = 9.78, range = 39–71). SSRT was therefore calculated by subtracting the mean SSD from the finishing time of the stop process. The finishing time was calculated by integrating the go reaction times (RTs) distribution as follows: (i) RTs of correct go trials in the stop condition were rank-ordered; (ii) the *n*th RT was selected (*n*th obtained from multiplying the number of go trials by the probability of responding to the stop signal); (iii) the SSD was subtracted from this *n*th RT. The SSD was averaged from the values for the last six moves in each of the three staircases. One of our main measures of interest was the switch RT, obtained between a difference score between switch RT minus Go RT in the switch condition. On this task, Go RTs in the switch condition are usually slower than Go RTs in stop condition due to a proactive “waiting” strategy that is often present in anticipation of a harder condition. To quantify this proactive and context-specific form of cognitive control, we calculated the response delay effect (RDE) [i.e., mean Go RTs (switch condition) – mean Go RTs (stop condition)]. Omission (absence of key pressing during a go trial) and discrimination errors (responding as the contrary arrow direction) were also of interest. Other measures were used to ensure that TMS pulses were not interfering with attention modulation in our participants (Go RTs and errors).

The main analyses were performed considering the independent variables as categorical [TBS (sham vs. IFG), pulses (no pulse vs. pulse), and locus (pre-SMA vs. vertex)] and the dependent variables as continuous (SSRT, RDE, Switch RT). Thus, a general lineal model (GLM) repeated measures procedure (2 × 2 × 2) was performed. Significant interactions were followed up using paired *t*-tests, corrected for multiple comparisons using Holm–Bonferroni corrections.

## Results

Two participants were excluded from the analysis because of uncomfortable sensory feelings whilst undergoing the cTBS IFG session. Data for the main variables of interest in stop and switch conditions of the task are presented in Table 1.

**Table 1:** **Stop and switch condition behavior results**.

	Sham TBS				IFG TBS			
	Pre-SMA		Vertex		Pre-SMA		Vertex	
	No pulse	Pulse	No pulse	Pulse	No pulse	Pulse	No pulse	Pulse
**STOP CONDITION**								
Go RT	459.43 (66.8)	466.61 (64.8)	450.01 (71.5)	443.82 (62.1)	471.74 (89.6)	471.49 (95.3)	465.94 (98.7)	469.20 (98.3)
Omission errors	2.28 (2.3)	2.78 (2.6)	2.5 (2.1)	2.80 (2.5)	3.07 (3.6)	1.92 (2.4)	3.14 (3.0)	2.92 (2.4)
SSRT	252.83 (36.5)	282.40 (41.4)	261.20 (33.8)	254.56 (27.9)	278.61 (36.0)	277.41 (52.6)	283.04 (44.9)	282.78 (40.4)
RDE	16.63 (21.1)	3.17 (24.9)	17.66 (30.1)	20.13 (24.4)	3.92 (29.6)	13.67 (32.2)	10.54 (19.7)	5.25 (22.4)
**SWITCH CONDITION**								
Go RT	473.21 (84.2)	471.21 (72.0)	475.05 (68.2)	468.67 (71.1)	481.03 (102.8)	478.01 (101.2)	476.48 (94.7)	474.46 (97.6)
Omission errors	0.50 (0.8)	0.64 (0.9)	0.35 (0.84)	0.71 (0.46)	0.78 (1.2)	1.42 (0.5)	0.57 (1.2)	1.35 (0.49)
Switch RT	27.92 (80.0)	30.68 (74.7)	23.49 (48.3)	25.84 (43.0)	12.67 (82.3)	18.18 (82.9)	31.51 (78.6)	32.06 (75.8)
Discrimination errors	1.21 (1.4)	3.00 (3.5)	0.57 (0.75)	2.92 (2.0)	1.21 (3.1)	1.35 (2.3)	1.14 (1.6)	2.64 (2.8)

*Means and standard deviations (in brackets) are given for each measure*.

### Stop condition I – reactive inhibition

**Figure 2:**
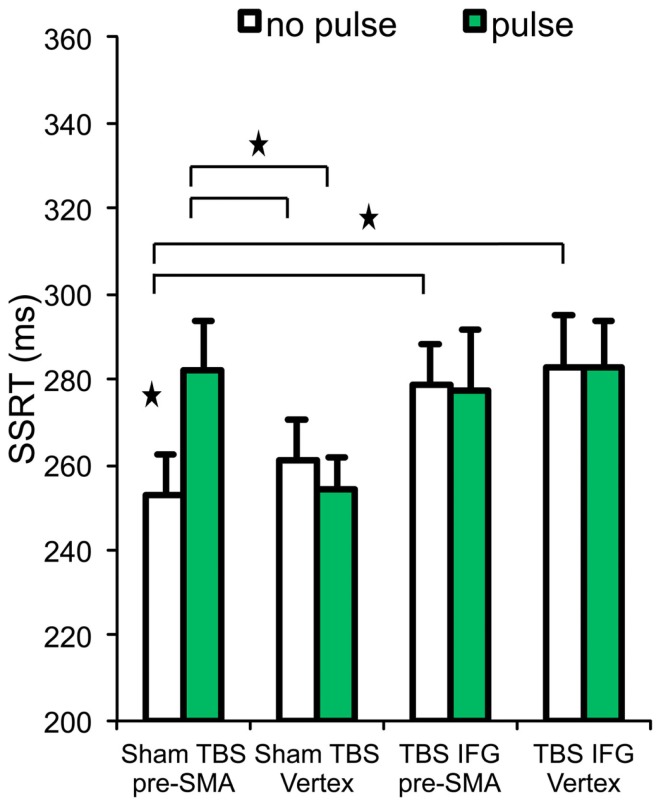
**Stop signal reaction time (SSRT) across the four experimental conditions**. Stars and horizontal bars represent significant differences (*p* < 0.05). Error bars indicate standard error of the mean.

Results relative to the SSRT are reported in Table 1 and Figure 2. Our initial hypothesis regarding the relevance of the right IFG in inhibition was confirmed. A significant interaction was found between TBS (sham vs. IFG) and locus (pre-SMA vs. vertex) [*F*_(1,13)_ = 4.74, *p* = 0.04]. Follow-up paired *t*-tests revealed shorter SSRTs after cTBS over the IFG compared to after sham cTBS [*t*_(13)_ = −2.25, *p* = 0.04]. This was also found in trials without pulses over the vertex [*t*_(13)_ = 2.99, *p* = 0.01].

To establish our prediction of the pre-SMA critical involvement in inhibition, a significant interaction between pulses (no pulse vs. pulse) and locus (pre-SMA vs. vertex) on SSRT [*F*_(1,13)_ = 7.54, *p* = 0.01] confirms our hypothesis. Moreover, follow-up paired *t*-tests revealed that SSRT was significantly longer on trials receiving pulses over pre-SMA (after sham cTBS) than those trials without pulses over the same region [*t*_(13)_ = −2.84, *p* = 0.01]. This result purely represents the effect of TMS pulses at 100 ms without any influence of cTBS. In the sham cTBS condition, SSRT was longer on trials with pulses over the pre-SMA compared to trials with pulses over the vertex [*t*_(13)_ = 3.29, *p* = 0.006] and also on trials without pulses in vertex [*t*_(13)_ = −2.66, *p* = 0.01] (Figure 2).

However, contrary to our expectations, when pulses were delivered to pre-SMA (while subjects were under cTBS effects over IFG), it did not produce significant differences on SSRT (Figure 2). No significant main effect of TBS (sham vs. IFG) [*F*_(1,13)_ = 2.35, *p* = 0.14] and interaction between TBS (sham vs. IFG) and pulses (no pulse vs. pulse) [*F*_(1,13)_ = 3.70, *p* = 0.07] were observed in SSRT.

### Stop condition II – proactive inhibition

**Figure 3:**
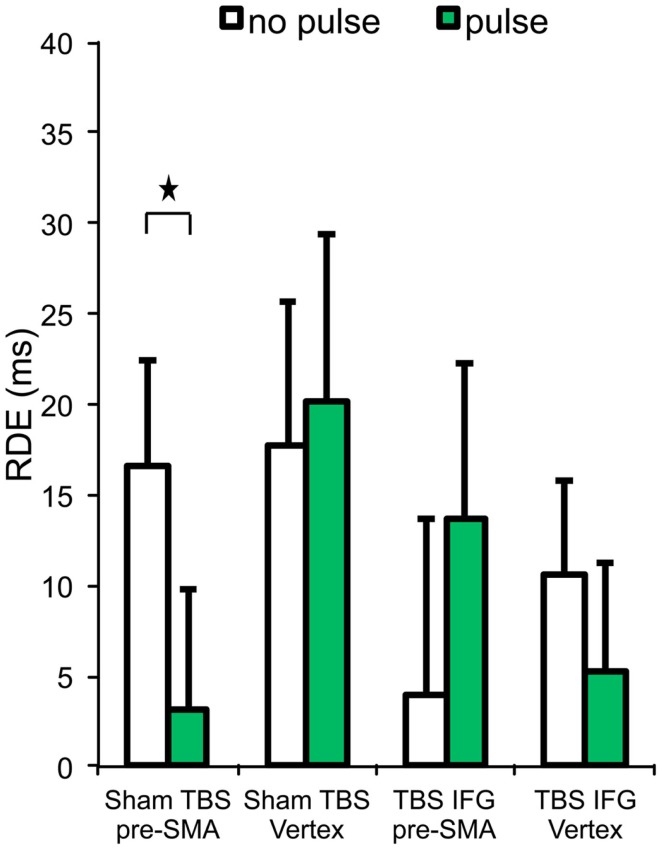
**Response delay effect (RDE) across the four experimental conditions**. Stars represent significant differences (*p* < 0.05). Error bars indicate standard error of the mean.

We wanted to further investigate how pre-SMA cognitive control functions are implemented in other aspects of the current behavior, such as in proactive inhibition. This behavior was measured using the RDE difference score. Results relative to the RDE are reported in Table 1 and Figure 3.

A significant interaction was found between cTBS (sham vs. IFG), pulses (no pulse vs. pulse), and locus (pre-SMA vs. vertex) on RDE [*F*_(1,13)_ = 6.49, *p* = 0.02]. In the sham cTBS condition, paired *t*-tests showed that RDE was significantly worse on trials receiving pulses over pre-SMA than those trials without pulses [*t*_(13)_ = 2.42, *p* = 0.03].

No significant main effect of TBS (sham vs. IFG) [*F*_(1,13)_ = 3.18, *p* = 0.09], interaction between TBS (sham vs. IFG) and pulses (no pulse vs. pulse) [*F*_(1,13)_ = 0.83, *p* = 0.37], or locus (pre-SMA vs. vertex) [*F*_(1,13)_ = 0.33, *p* = 0.57] were observed in RDE.

### Switch condition

Results relative to the switching condition are reported in Table 1. No significant main effect of TBS (sham vs. IFG) [*F*_(1,13)_ = 0.06, *p* = 0.80], pulses [*F*_(1,13)_ = 0.26, *p* = 0.61], or the interaction between TBS (sham vs. IFG) and pulses (no pulse vs. pulse) [*F*_(1,13)_ = 0.01, *p* = 0.96], or locus (pre-SMA vs. vertex) [*F*_(1,13)_ = 0.63, *p* = 0.43] were found in the switching condition.

The significant effects of pulses over pre-SMA on SSRT and RDE were unlikely due to lapses of attention or TMS-related discomfort, indicated by no significant interactions between locus and pulses for Go RTs and error generation for both stop and switching conditions (all values *p* > 0.3).

## Discussion

We used a combined *online* and *offline* TMS approach to show that *online* stimulation of the right pre-SMA exclusively altered two types of inhibition (reactive and proactive inhibition), but did not produce major effects on switching behavior. Importantly, after cTBS over the right IFG, stopping behavior was worse as compared to sham cTBS. The results support the notion that both the pre-SMA and IFG are key areas during cognitive control operations. The current study provides evidence to suggest that the right pre-SMA exerts an inhibitory function when switching of actions is needed.

### The stopping process

We found that *online* TMS pulses over the right pre-SMA (after sham cTBS) altered the stopping process (prolonged SSRT), but also altered the preparation to inhibit [as reflected by a reduction in response times needed to adapt between different task conditions during proactive inhibition (shorter RDE)]. We first consider potential reasons for the finding of slowed reactive inhibition after pulses over the pre-SMA.

In our study, similar to Chen et al. (2009), TMS pulses were applied 100 ms after stimulus presentation and interrupted reactive inhibition. Similarly, when using *online* TMS over right (Cai et al., 2012) or left pre-SMA (Chen et al., 2009), our results are similar to that reported previously; the inhibition of actions were delayed compared to control stimulation. In Chen et al. (2009), two repeated pulses (0 and 100 ms) were applied after a stimulus and in Cai et al. (2012) different pulse intervals (125/150 and 175/200 ms) were employed. Their approach may cover the whole inhibitory response process since more pulses were applied. These authors did not find a pulse timing effect, suggesting that they altered the whole implementation of stopping. Since it is believed that other regions will follow pre-SMA activity to exert stopping action (Neubert et al., 2010), such as M1, our TMS pulses probably delayed the overall process. In the current study, the pulses may have interfered with the early part of the adjustment of stopping behavior and therefore, the subsequent inhibition implementation requires some extra milliseconds to occur.

The use of a combined stop and switch task allowed us to obtain a measure of response inhibition during adaptation between both conditions, and separates a rapid form of inhibition (reactive inhibition) from response modulation to expected stopping behavior (proactive inhibition). Proactive control is required in a sequence of two different actions, in which one is able to predict upcoming stimulus to exert a potential behavioral change in order to guarantee goal-directed behavior. It is guided by endogenous signals, present along the whole action-execution process which guide the behavioral outcome. This requires a working memory element to sustain activity before the action is executed. Longer times taken to choose between two available actions will increase the likelihood of success. We found worse response adaptation (as revealed by slower time adjustment between go trials of both arrow directions) on trials with TMS pulse over pre-SMA than on trials without pulses in the sham cTBS condition, suggesting that pre-SMA also exerts modulation of inhibitory behavior. Probably, the pre-SMA exerts its proactive role across the entire response adaptation process. Our hypothesis fits well with direct single-unit recordings in the pre-SMA of monkeys that revealed neuronal activity during stopping, but also when analyzing trial history during non-stop trials (Stuphorn and Emeric, 2012).

Imaging evidence indicates that the DLPFC participates in mediating response modulation (Mostofsky et al., 2003; Jahfari et al., 2010; Criaud and Boulinguez, 2012). The task used here requires online monitoring of relevant cues to adapt ones behavior between conditions. Thus, in the current study, working memory is relevant and we consider that pre-SMA stimulation could have indirectly affected DLPFC activation during active engagement during different conditions. Moreover, in a recent meta-analysis that investigated studies using the go no-go task, pre-SMA activity was proposed as being important for attentional and working memory elements of behavior (Criaud and Boulinguez, 2012). They report that in complex tasks, where participants need to engage in a certain frequency pattern of responses, the pre-SMA, as well as the DLPFC and other frontal regions, are activated. Therefore, it seems plausible to link our results in the role of pre-SMA during proactive inhibition with perhaps a working memory influence coming from DLPFC when adaptation between two ongoing behaviors is needed.

Importantly, firing in the pre-SMA was present in monkeys during both stop trials and non-stop trials (Stuphorn and Emeric, 2012). During non-stop trial history, firing peaks were observed that suggested that the pre-SMA could induce proactive inhibition throughout phasic firing due to its reactive function. In our study we observed significant pre-SMA involvement in both reactive and proactive inhibition behaviors. Therefore, it could be possible that firing of the pre-SMA may use several phasic bursts (important in reactive inhibition) to produce general proactive behaviors. Thus, it makes sense that a meeting point is shared within the pre-SMA during fast and reactive inhibition of actions and during more prolonged proactive inhibitory ones.

### Understanding the role of the pre-SMA in cognitive control

One main objective of this study was to dissociate cognitive control functions within the pre-SMA. We tested how participants switched behavior by producing a new movement and inhibiting the initial one. This behavior was not affected when participants received *online* TMS pulses (compared to no TMS pulses) over the pre-SMA in both cTBS conditions. This negative result may indicate that either the pre-SMA is not taking part in switching to new behaviors, or that this process entails a different time-period that our protocol was unable to interfere with. Amongst the functions often attributed to the pre-SMA, such as response inhibition, shifting from automatic to volitional actions, monitoring of action, or conflict resolution (Rushworth et al., 2002, 2005; Kennerley et al., 2004; Wittfoth et al., 2006; Aron et al., 2007; Isoda and Hikosaka, 2007; Nachev et al., 2007; Jaffard et al., 2008), our intention was to discern between inhibition and switching of actions. A role for the pre-SMA in conflict resolution has been discredited in two TMS studies (Obeso et al., 2011; Cai et al., 2012). Thus, inhibition and switching seems to cooperate and may act in parallel during conflict resolution (Nachev et al., 2008; Kenner et al., 2010).

In one earlier study that applied rTMS over the pre-SMA, impairment in a response selection task was specific to a switching a condition and not during other trial types (Rushworth et al., 2002). However, the authors used a train of TMS pulses, which may have transiently affected pre-SMA and disrupted switching behavior across later time-windows. We could only interfere at one specific time-window (100 ms). Also, one fMRI study (Kenner et al., 2010) that used a combined switching and stop signal task similar to that employed here, showed a common neural recruitment during both inhibition and switching trials, mainly involving the known stopping network. Their data suggests that switching is sustained by the same mechanisms as inhibition. Also, since the IFG was highlighted in their imaging results, applying cTBS here should have also affected switching in our participants. The lack of findings to support this could be explained by alternative IFG functions during switching, such as stimulus detection (Hampshire et al., 2010; Verbruggen et al., 2010; Lenartowicz et al., 2011). Thus, our TMS protocol (both *online* or *offline* approach) should have interfered with action switching through perturbation of inhibitory-related functions in pre-SMA or IFG. However, since our approach may induce interaction effects between cTBS and online TMS (Silvanto and Pascual-Leone, 2008), additional conditions could provide further information on each regions role in response switching. Also, failure to observe such changes are likely due to methodological constraints, such as target location (Verbruggen et al., 2010) or TMS coil distance (Ruohonen and Ilmoniemi, 2002).

### Reactive vs. proactive inhibitory model

How brain regions use cognitive control to regulate behavior can be understood in a simplified inhibitory model (Aron, 2011). Based on human and animal data, the inhibitory model suggests that a signal sent from the IFG to the STN, via the hyperdirect pathway, will promote fast and sudden inhibition of actions (reactive inhibition). However, DLPFC projects to the striatum, via the indirect pathway to foster response modulation in order to respond to stopping stimuli (proactive inhibition). DLPFC is important for working memory (Petrides and Pandya, 1999; Muller and Knight, 2006). Since stopping in response to upcoming stimuli requires working memory, and response inhibition studies report DLPFC activation (Mostofsky et al., 2003; Jahfari et al., 2010), it could contribute to response adaptation and proactive inhibition in our task. However, our findings give credence to the hyperdirect pathway hypothesis relative to reactive inhibition.

Some data suggests that reactive inhibition can be controlled by the hyperdirect pathway between the IFG and the STN (Aron et al., 2007; Jahfari et al., 2011). Here, after the sham cTBS condition, TMS pulses changed inhibitory performance of our participants, but to a similar degree when real cTBS was applied over the IFG. This may suggest that both the IFG and the pre-SMA are important for the implementation of inhibition. Interpretation according to the model should proceed with care, but our data is consistent with an important role of the hyperdirect pathway involving the IFG.

In addition to the previous point, the inhibitory model (Aron, 2011) could benefit from the viewing of the pre-SMA as a modulatory structure during proactive inhibition when different response types are available. The model proposes as key structures the DLPFC and caudate, but not the pre-SMA. Based on previous evidence (Forstmann et al., 2008; Jaffard et al., 2008; Boulinguez et al., 2009; Chikazoe et al., 2009; Chen et al., 2010; Zandbelt and Vink, 2010; Stuphorn and Emeric, 2012), when an action needs to be adapted throughout time, a modulatory role of the pre-SMA over the striatum (via the indirect pathway) may promote proactive inhibition. Indeed, during preparation of an upcoming stop signal, the pre-SMA exerts a modulatory role (Zandbelt and Vink, 2010; Swann et al., 2012) as well as increased striatal activity as a function of stop signal probability (Li et al., 2008; Chao et al., 2009; Zandbelt and Vink, 2010). Supporting this view, one animal study reported changes in proactive behavior after medial PFC stimulation (Stuphorn and Schall, 2006). Considering the above, we suggest that the pre-SMA sends inputs to striatum to inhibit external globus pallidus (GPe), which will release the internal globus pallidus (GPi) to send inhibitory signals to the pre-SMA or M1. Thus, the current results may reflect an altered efference from the striatum that in turn will induce a blockade over GPi to send back, at the appropriate time, signals to the cortex. While this hypothesis does not exclude a potential role of working memory functions of the DLPFC during proactive inhibition, it gives causal TMS evidence to a role for the pre-SMA.

## Summary

Overall, our results provide further understanding of the role of the pre-SMA during stopping behavior. The network requires intact contribution of its regions (IFG, pre-SMA) to operate successfully. We propose a dissociation between switching and inhibitory-related functions in pre-SMA, since we show data to support the role of the pre-SMA as an inhibitory structure. We further confirm that pre-SMA exerts a relevant role in reactive inhibition, but we also demonstrate its role during proactive inhibition.

## Conflict of Interest Statement

The authors declare that the research was conducted in the absence of any commercial or financial relationships that could be construed as a potential conflict of interest.
